# Agrawal Axisymmetric Rotational Stagnation-Point Flow of a Water-Based Molybdenum Disulfide-Graphene Oxide Hybrid Nanofluid and Heat Transfer Impinging on a Radially Permeable Moving Rotating Disk

**DOI:** 10.3390/nano12050787

**Published:** 2022-02-25

**Authors:** Umair Khan, Aurang Zaib, Anuar Ishak, Iskandar Waini, Abdel-Haleem Abdel-Aty, Mikhail A. Sheremet, Ibrahim S. Yahia, Heba Y. Zahran, Ahmed M. Galal

**Affiliations:** 1 Department of Mathematical Sciences, Faculty of Science and Technology, Universiti Kebangsaan Malaysia, UKM, Bangi 43600, Selangor, Malaysia; umairkhan@iba-suk.edu.pk (U.K.); anuar_mi@ukm.edu.my (A.I.); 2 Department of Mathematics and Social Sciences, Sukkur IBA University, Sukkur 65200, Sindh, Pakistan; 3 Department of Mathematical Sciences, Federal Urdu University of Arts, Science & Technology, Gulshan-e-Iqbal Karachi 75300, Sindh, Pakistan; aurangzaib@fuuast.edu.pk; 4 Fakulti Teknologi Kejuruteraan Mekanikaldan Pembuatan, Universiti Teknikal Malaysia Melaka, Hang Tuah Jaya, Durian Tunggal 76100, Melaka, Malaysia; iskandarwaini@utem.edu.my; 5 Department of Physics, College of Sciences, University of Bisha, P.O. Box 344, Bisha 61922, Saudi Arabia; amabdelaty@ub.edu.sa; 6 Physics Department, Faculty of Science, Al-Azhar University, Assiut 71524, Egypt; 7 Laboratory on Convective Heat and Mass Transfer, Tomsk State University, 634050 Tomsk, Russia; 8 Laboratory of Nano-Smart Materials for Science and Technology (LNSMST), Department of Physics, Faculty of Science, King Khalid University, P.O. Box 9004, Abha 61413, Saudi Arabia; isyahia@gmail.com (I.S.Y.); heldemardash@kku.edu.sa (H.Y.Z.); 9 Research Center for Advanced Materials Science (RCAMS), King Khalid University, P.O. Box 9004, Abha 61413, Saudi Arabia; 10 Nanoscience Laboratory for Environmental and Biomedical Applications (NLEBA), Metallurgical Lab. 1, Department of Physics, Faculty of Education, Ain Shams University, Roxy, Cairo 11757, Egypt; 11 Mechanical Engineering Department, College of Engineering, Prince Sattam Bin Abdulaziz University, Wadiad Dawaser 11991, Saudi Arabia; ahm.mohamed@psau.edu.sa; 12 Production Engineering and Mechanical Design Department, Faculty of Engineering, Mansoura University, Mansoura 35516, Egypt

**Keywords:** rotating flow, hybrid nanofluid, moving rotating disk, multiple solutions

## Abstract

The hybrid nanofluid has sparked new significance in the industrial and engineering sectors because of their applications like water heating in solar and analysis of heat exchanger surfaces. As a result, the current study emphasizes the analysis of heat transfer and Agrawal axisymmetric flow towards a rotational stagnation point incorporated via hybrid nanofluids imposing on a radially permeable shrinking/stretching rotating disk. The leading partial differential equations are refined into ordinary differential equations by using appropriate similarity variables. The bvp4c solver in MATLAB is then employed to solve the simplified system numerically. The current numerical procedure is adequate of generating double solutions when excellent initial guesses are implemented. The results show that the features of fluid flow along with heat transfer rate induced by hybrid nanofluid are significantly influenced. The Nusselt number and the tendency of the wall drag force can be improved as the concentration of nanoparticles and the suction factor are increased. Moreover, the results of the model have been discussed in detail for both solution branches due to the cases of rotating disk parameter as well as non-rotating disk parameter. Therefore, an extraordinary behavior is observed for the branch of lower solutions in the case of rotating disk parameter. In addition, the shear stress in the radial direction upsurges for the first solution but declines for the second solution with higher values of suction. Moreover, the rotating parameter slows down the separation of the boundary layer.

## 1. Introduction

Several researchers are keen to investigate the nanofluid (the combining of nanoparticles in convectional fluids) because of the remarkable progress in modern sciences and nanotechnology. The research of Choi and Eastman [1] demonstrated that copper nanoparticles aid to uplift the heat transfer performance. Nanofluids have been recognized as possible thermo-fluids for potential developments due to their fascinating convective thermophysical behavior [2,3]. The unusual properties of nanofluids have received widespread recognition in a variety of medical, engineering, and industrial applications such as the efficiency of diesel generators, nuclear reactors, engine cooling, micro-manufacturing, cancer treatment, solar liquid heating, and various sorts of heat exchangers [4,5,6]. Bachok et al. [7] inspected the 3D flow towards a stagnation point induced by nanofluid by considering three distinct nanoparticles. The mass and heat transfer through an annulus in a porous media with non-Newtonian fluid was scrutinized by Ellahi et al. [8]. Khan et al. [9] explored the 3D flow and heat transfer induced by nanofluid in two opposed directions through a stretchable horizontal plane surface. Some important investigations regarding nanofluid with different aspects can be observed in [10,11,12,13,14].

Recently, scattering composite nano-powder or different nanomaterials with sizes or bulks ranging from 1 to 10 nm in the convectional fluid resulted in the development of new-fangled engineered nanofluids [15]; which is recognized as a hybrid nanoliquid (HN). The HN is a contemporary fluid that has the potential to develop the performance of heat transfer and thermal physical features. Sarkar et al. [16] and Sidik et al. [17] reported advancements on hybrid nanofluid processing techniques, thermophysical characteristics of HNs, and recent applications of HNs. Huminic and Huminic [18] emphasized the importance of hybrid nanofluids in different applications like heat pipes, plates, air cooling systems, tabular heat exchangers, and tube and shell heat exchangers. Yousefi et al. [19] conducted a review of the curly or wavy cylinder comprising the substantial consequence of HN and flow of stagnation-point (SPF). The unsteady flow and heat transport induced by a hybrid nanoliquid over a shrinkable/stretchable sheet was examined by Waini et al. [20] and they presented double solutions as well as performed stability analysis. Kumar et al. [21] inspected the impact of erratic heat sink/source on the magneto thin-film flow subject to ferrite hybrid nanofluid through an elongating sheet. Khan et al. [22] discussed the influence of thermal stratification on the magneto fluid flow by involving hybrid nanofluid of single and multi-wall CNTs through a slender stretching surface. Kolsi et al. [23] inspected the slip flow towards a stagnation-point induced by ethylene glycol-based hybrid nanofluid through a stretchable cylinder. They observed that the heat transfer rate improves for the non-convective stretchable cylinders. Zhang et al. [24] examined the impact of the induced magnetic effect on the flow with hybrid nanofluid through a stretchy surface. Recently, Khan et al. [25] considered the non-Newtonian fluid to scrutinize the properties of HN near a stagnation point comprising different shape factors as well as base fluids.

The scrutiny of the flow near a stagnation point through a rotated disc has attracted a lot of attention over the last decade or so. Hiemenz [26] found an exact solution of Navier-Stokes equations, which describe 2D stable flow addressed orthogonally at a smooth infinite plate. Soon after, Homann [27] expanded this problem to incorporate the case of an axisymmetric flow. Hannah [28] extended Homann’s [27] classic SPF on a flat sheet through the irrotational far field to flow against a rotating disc. However, Agrawal [29] developed a novel axial symmetric stagnation-point flow subject to external rotating flow and impact normal to a plane infinite wall. Later on, this problem has been extended numerically by Weidman [30]. Turkyilmazoglu [31] examined the 3D rotating flow near an MHD stagnation-point through a stretchable radially rotating disc. He observed that the all effects like magnetic, rotation, and stretching parameters have a significant impact on the shear stresses and stagnation velocities. Weidman [32,33] examined the Agrawal axisymmetric flow towards a stagnation-point past a flat surface and stretching surface, respectively. Lately, Lok et al. [34] extended the research of Weidman [33] by considering the axisymmetric rotational flow towards a stagnation-point imposing a rotated porous stretchable/shrinkable disk and presented double solutions.

Encouraged by the preceding research, the novelty of this study is to explore the properties of a rotational Agrawal axisymmetric flow subject to a shrinkable or stretchable porous rotating disk near a stagnation-point comprising the significant influence of hybrid nanofluid. The dual solutions for the cases of rotating disk parameter and non-rotating disk parameter are also one of the novelties or key objectives of this given analysis. Following [33], the problem has been reduced to a similarity appearance containing different pertaining parameters. Numerical solutions for prominent values of the comprised influential constraints are procured, demonstrating the existence of double solutions providing additional insight into the nature of the considered problem. The paper arrangement is as follows: The problem formulation is demonstrated in Section 2. Section 3 reports the numerical results computations of the leading equations and Section 4 concludes the paper with a discussion and closing remarks.

## 2. Materials and Methods

**Figure 1:**
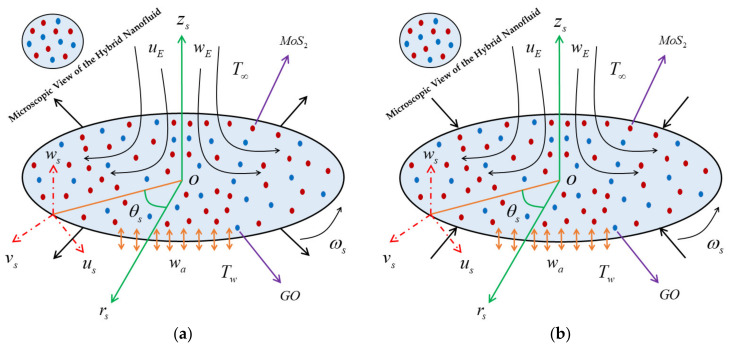
The physical geometry of the problem: (**a**) Rotating stretching disk, (**b**) Rotating shrinking disk.

The overall mechanism of the Agrawal flow configuration of the problem is schematically shown in Figure 1, where the analysis of heat transfer near an axisymmetric rotational stagnation-point flow induced by hybrid nanofluid impinging radially a permeable moving rotating disk are contemplated. The problem is initially expressed in terms of cylindrical coordinates (*r_s_*, θ*_s_*, *z_s_*) considered in the following radial, azimuthal, and axial directions, respectively, with the associated component of velocities (*u_s_*, *v_s_*, *w_s_*). The Agrawal flow is symmetric to the *r_s_*, θ*_s_*–plane and also axisymmetric about the axial direction (*z_s_*-axis), i.e., the variation along the coordinate θ*_s_* (azimuthal direction) is ignored. The stagnation line is at *z_s_* = 0 and the domain of the flow is in the upper half-plane. Therefore, the stretching/shrinking disk is rotating about the axial direction (*z_s_*-axis) together with a fixed angular velocity ω*_s_*. In addition, the hybrid nanofluid is composed of two dissimilar nanoparticles such as graphene oxide (GO) and molybdenum disulfide (MoS_2_) nanoparticles along with normal fluid. The hybrid nanomaterials physical features are presumed to be in the form of equilibrium, and there is no-slip occurs between them. Further, it is supposed that the module of the ambient velocities is considered via $u_{E}\left(r_{s},z_{s}\right)=2ar_{s}z_{s}$, $v_{E}\left(r_{s},z_{s}\right)=0$ and $w_{E}\left(r_{s},z_{s}\right)=-2az_{s}^{2}$, where *a* is a constant factor measuring the strength of the Agrawal flow having units (LT)^–1^, see Weidman [33]. Moreover, the surface velocity at the wall *z_s_* = 0 is denoted by $u_{w}=a^{2/3}\upsilon_{f}^{1/3}r_{s}$ and $v_{w}=\omega_{s}r_{s}$, where ω*_s_* is the fix rotational velocity of the disc, while the uniform transparent velocity through the wall is implemented via *w_a_*, where *w_a_* < 0 for suction and *w_a_* > 0 for blowing of the fluid. The surface and the free stream constant temperatures are indicated by *T_w_* and *T*_∞_, respectively with *T_w_* > *T*_∞_. Using the above mentioned assumptions, the modeled requisite governing PDEs equations are premeditated by (see Weidman [33]):

(1) $$\frac{\partial u_{s}}{\partial r_{s}}+\frac{u_{s}}{r_{s}}+\frac{\partial w_{s}}{\partial z_{s}}=0,$$

(2) $$u_{s}\frac{\partial u_{s}}{\partial r_{s}}-\frac{v_{s}{}^{2}}{r_{s}}+w_{s}\frac{\partial u_{s}}{\partial z_{s}}=-\frac{1}{\rho_{hnf}}\frac{\partial P_{s}}{\partial r_{s}}+\frac{\mu_{hnf}}{\rho_{hnf}}\left(\frac{\partial^{2}u_{s}}{\partial r_{s}{}^{2}}+\frac{1}{r_{s}}\frac{\partial u_{s}}{\partial r_{s}}-\frac{u_{s}}{r_{s}{}^{2}}+\frac{\partial^{2}u_{s}}{\partial z_{s}{}^{2}}\right),$$

(3) $$u_{s}\frac{\partial v_{s}}{\partial r_{s}}+\frac{u_{s}v_{s}}{r_{s}}+w_{s}\frac{\partial v_{s}}{\partial z_{s}}=\frac{\mu_{hnf}}{\rho_{hnf}}\left(\frac{\partial^{2}v_{s}}{\partial r_{s}{}^{2}}+\frac{\partial }{\partial r_{s}}\left(\frac{v_{s}}{r_{s}}\right)+\frac{\partial^{2}v_{s}}{\partial z_{s}{}^{2}}\right),$$

(4) $$u_{s}\frac{\partial w_{s}}{\partial r_{s}}+w_{s}\frac{\partial w_{s}}{\partial z_{s}}=-\frac{1}{\rho_{hnf}}\frac{\partial P_{s}}{\partial z_{s}}+\frac{\mu_{hnf}}{\rho_{hnf}}\left(\frac{\partial^{2}w_{s}}{\partial r_{s}{}^{2}}+\frac{1}{r_{s}}\frac{\partial w_{s}}{\partial r_{s}}+\frac{\partial^{2}w_{s}}{\partial z_{s}{}^{2}}\right),$$

(5) $$u_{s}\frac{\partial T_{s}}{\partial r_{s}}+w_{s}\frac{\partial T_{s}}{\partial z_{s}}=\frac{k_{hnf}}{{\left(\rho c_{p}\right)}_{hnf}}\left(\frac{\partial^{2}T_{s}}{\partial r_{s}{}^{2}}+\frac{1}{r_{s}}\frac{\partial T_{s}}{\partial r_{s}}+\frac{\partial^{2}T_{s}}{\partial z_{s}{}^{2}}\right),$$

along with a subject to the boundary conditions (BCs) are:

(6) $$\left.\begin{array}{l} u_{s}=\lambda u_{w},\;w_{s}=w_{a},\;v_{s}=v_{w},\;T_{s}=T_{w}\;\mathrm{at}\;z_{s}=0,\\ \frac{\partial u_{s}}{\partial z_{s}}\to \frac{\partial u_{E}}{\partial z_{s}},\;v_{E}\to 0,\;T_{s}\to T_{\infty }\;,\;w_{s}\to w_{e}=-2az_{s}{}^{2}\;as\;z_{s}\to \infty . \end{array}\right\}$$

In the above governing equations, *u_s_*, *v_s_* and *w_s_* are the component of velocities along *r_s_*, θ*_s_* and *z_s_*-essential coordinate axes, *P_s_* is the pressure, *T_s_* is the temperature, and λ is the constant stretching/shrinking parameters with λ > 0 for the stretching sheet, λ < 0 for the shrinking sheet, and λ = 0 for the static disk. Further, ρ*_hnf_* is the density, *k_hnf_* is the thermal conductivity, ${(\rho c_{p})\;}_{hnf}$ is the heat capacity, and μ*_hnf_* is the dynamic viscosity of the hybrid nanofluid, which is given as (see [35,36,37]).

(7) $$\left\{\begin{array}{cc} \; & \frac{\mu_{hnf}}{\mu_{f}}=\left(1-\varphi \right){\;}^{-2.5},\;\;\;\;\mathrm{where}\;\;\;\;\;\varphi =\varphi_{a}+\varphi_{b},\\ \; & \frac{\rho_{hnf}}{\rho_{f}}=\varphi_{a}\left(\frac{\rho_{a}}{\rho_{f}}\right)+\varphi_{b}\left(\frac{\rho_{b}}{\rho_{f}}\right)+\left(1-\varphi \right),\\ \; & \frac{k_{hnf}}{k_{f}}=\left(C_{a}+C_{b}\right)\times {\left(C_{a}+C_{c}\right)}^{-1},\;\;\mathrm{where}\;\;C_{a}=\frac{\left(\varphi_{a}k_{a}+\varphi_{b}k_{b}\right)}{\varphi },\\ \; & C_{b}=2k_{f}+2\left(\varphi_{a}k_{a}+\varphi_{b}k_{b}\right)-2\varphi k_{f},\;C_{c}=2k_{f}-\left(\varphi_{a}k_{a}+\varphi_{b}k_{b}\right)+\varphi k_{f}\\ \; & \frac{{\left(\rho c_{p}\right)}_{hnf}}{{\left(\rho c_{p}\right)}_{f}}=\varphi_{a}\left(\frac{{\left(\rho c_{p}\right)}_{a}}{{\left(\rho c_{p}\right)}_{f}}\right)+\varphi_{b}\left(\frac{{\left(\rho c_{p}\right)}_{b}}{{\left(\rho c_{p}\right)}_{f}}\right)+\left(1-\varphi \right), \end{array}\right.$$

where ϕ corresponds to the volume fraction of nanoparticle and is equal to the combination or sum of the two dissimilar nanomaterials such as ϕ*_a_* signifies to molybdenum disulfide nanoparticle (MoS_2_), ϕ*_b_* signifies to graphene oxide nanoparticle (GO), and ϕ = 0 indicates to a pure working fluid. Further, ρ*_a_*, ρ*_b_*, ρ*_f_*, *k_a_*, *k_b_*, *k_f_*, ${\left(\rho c_{p}\right)}_{a}$, ${\left(\rho c_{p}\right)}_{b}$ and ${\left(\rho c_{p}\right)}_{f}$ are the respective density, thermal conductivity and heat capacitance of the volume fraction of hybrid nanoparticle and the pure working fluid, respectively. In addition, the thermophysical properties of the working fluid and the two distinct nanomaterials are written in Table 1.

To simply the given problem further for the analysis, here, we let the following similarity dimensionless transformations which can be written as (see Weidman [34]):

(8) $$\begin{array}{l} u_{s}\left(r_{s},z_{s}\right)=a^{2/3}\upsilon_{f}^{1/3}r_{s}F^{\prime }\left(\xi \right),\;v_{s}\left(r_{s},z_{s}\right)=\omega_{s}r_{s}G\left(\xi \right),\xi ={\left(a/\upsilon_{f}\right)}^{1/3}z_{s},\\ S\left(\xi \right)=\frac{T_{s}-T_{\infty }}{T_{w}-T_{\infty }},\;w_{s}\left(r_{s},z_{s}\right)=-2a^{1/3}\upsilon_{f}^{2/3}F\left(\xi \right). \end{array}$$

Here, the primes indicate the respective derivative w.r.t to the symbols ξ and additionally the current expressions (8) lead us to yield

(9) $$w_{a}=-2a^{1/3}\upsilon_{f}^{2/3}f_{s}.$$

In aforesaid Equation (9), the notation *f_s_* reveals the mass flux factor with *f_s_* > 0 and *f_s_* < 0 corresponding to suction and as well as blowing, respectively, whereas *f_s_* = 0 is for an impermeable surface of the disk.

Now plugging the Equation (8) in the leading governing equations, where Equation (1) is identically satisfied while the remaining set of the equations are transfigured to the following ODEs as follows:

(10) $$\frac{\mu_{hnf}/\mu_{f}}{\rho_{hnf}/\rho_{f}}F^{\prime \prime \prime }+2{FF}^{\prime \prime }-F^{\prime 2}+\alpha_{A}G^{2}=0,$$

(11) $$\frac{\mu_{hnf}/\mu_{f}}{\rho_{hnf}/\rho_{f}}G\prime \prime +2\left(G^{\prime }F-GF^{\prime }\right)=0,$$

(12) $$\frac{1}{Pr}\frac{k_{hnf}/k_{f}}{{\left(\rho c_{p}\right)}_{hnf}/{\left(\rho c_{p}\right)}_{f}}S\prime \prime +2FS^{\prime }=0,$$

with appropriate BCs are:

(13) $$\begin{array}{l} F\left(0\right)=f_{s},\;F^{\prime }\left(0\right)=\lambda ,\;G\left(0\right)=1,\;S\left(0\right)=1,\\ F\prime \prime \left(\xi \right)\to 2,\;G\left(\xi \right)\to 0,\;S\left(\xi \right)\to 0\;\mathrm{as}\;\xi \to \infty . \end{array}$$

The aforesaid constructed similarity set of equations contain distinct distinguished parameters which are symbolically and namely demarcated as like $\alpha_{A}=\omega_{s}^{2}/a^{4/3}\upsilon_{f}^{2/3}$ resembles to the rotating disk constraint, dictating that $\alpha_{A}\geq 0$ and $Pr=\mu_{f}{\left(c_{p}\right)}_{f}/k_{f}$ the Prandtl number.

The physical quantities of practical interest or gradients are the wall drag forces in radial and azimuthal directions denoted via $C_{fr_{s}}$ and $C_{f\theta_{s}}$ while the heat transfer $Nu_{r_{s}}$ are defined as:

(14) $$C_{fr_{s}}=\frac{\mu_{hnf}}{\rho_{f}u_{w}{}^{2}}{\left.\left(\frac{\partial u_{s}}{\partial z_{s}}\right)\right|}_{z_{s}=0},\;C_{f\theta_{s}}=\frac{\mu_{hnf}}{\rho_{f}v_{w}{}^{2}}{\left.\left(\frac{\partial v_{s}}{\partial z_{s}}\right)\right|}_{z_{s}=0},\;Nu_{r_{s}}=-\frac{r_{s}k_{hnf}}{k_{f}\left(T_{w}-T_{\infty }\right)}{\left.\left(\frac{\partial T_{s}}{\partial z_{s}}\right)\right|}_{z_{s}=0}.$$

Exercising the similarity transformation (8) into (14), one obtains the form as:

(15) Rers1/2Cfrs=μhnfμfF″0, Rers1/2uwvwCfθs=αA−1μhnfμfG′0, Rers−1/2Nurs=−khnfkfS′0.

where Rers=uwrsυf is the local Reynolds number.

## 3. Numerical Procedure of the Multiple Solutions

This section of the work displays the detailed procedure of the numerical scheme (branches of first solution and second solution) along with the appropriate comparison of the code as shown in the form of a table. The similarity Equations (10)–(12) in conjunction with the border ailment (13) are extremely nonlinear and quite complicated to deal with it analytically or exactly. Therefore, the numerical scheme bvp4c was implemented to crush the system of similarity equations and find the outcomes for the first and lower branch solutions as shown in the form of a table and various distinct graphs. The procedure of the code was further based on a finite difference scheme that practices the Lobatto IIIa collocation formula to produce a *C*^1^–continuous solution. In addition, to start working on our solution procedure, the system of higher-order similarity equations was reduced to the requisite first-order via letting the new-fangled variables. To do the working process, let the new-fangled variables are:

(16) $$F=Z_{a},F^{\prime }=Z_{b},F^{\prime \prime }=Z_{c},G=Z_{d},G^{\prime }=Z_{e},S=Z_{f},S^{\prime }=Z_{g}$$

To get the system of first-order ODEs, therefore, we need to use the above Equation (16) in the dimensionless similarity Equations (10)–(13), one obtains the following form as follows:

(17) $$\frac{d}{d\xi }\left(\begin{array}{l} Z_{a}\\ Z_{b}\\ Z_{c}\\ Z_{d}\\ Z_{e}\\ Z_{f}\\ Z_{g} \end{array}\right)=\left(\begin{array}{l} Z_{b}\\ Z_{c}\\ \frac{\rho_{hnf}/\rho_{f}}{\mu_{hnf}/\mu_{f}}\left(Z_{b}^{2}-2Z_{a}Z_{c}-\alpha_{A}Z_{d}^{2}\right)\\ Z_{e}\\ \frac{\rho_{hnf}/\rho_{f}}{\mu_{hnf}/\mu_{f}}\left(2Z_{d}Z_{b}-2Z_{e}Z_{a}\right)\\ Z_{g}\\ -2\frac{Pr{\left(\rho c_{p}\right)}_{hnf}/{\left(\rho c_{p}\right)}_{f}}{k_{hnf}/k_{f}}Z_{g}Z_{a} \end{array}\right)$$

subject to the appropriate ICs are:

(18) $$\left.\begin{array}{l} Z_{a}\left(\xi \right)=f_{s},\;Z_{b}\left(\xi \right)=\lambda ,\;Z_{d}\left(\xi \right)=1,\;Z_{f}\left(\xi \right)=1\;\mathrm{as}\;\xi =0\\ Z_{c}\to 2,\;Z_{d}\to 0,\;Z_{f}\left(0\right)\to 0\;\mathrm{as}\;\xi \to \infty . \end{array}\right\}$$

The residuality of outcomes controls the error and mesh selection. It is safe to say that this is a residual-type technique, whose efficiency relies on the initial early guesses provided as well as the satisfaction of the ICs (initial conditions 18). Moreover, the border of the boundary layer thickness ξ = ξ_max_ is taken to be a fixed finite value four which can hold the border ailments (13) asymptotically while the mesh-size selection is around 0.01. As a result, the length-size fluctuations are employed to achieve higher accuracy or correctness equal to a level of 10^−10^. Furthermore, the current model has two dissimilar branch solutions. Therefore, the code needed two different guesses for the outcome of each branch. The guess for the first solution is quite easier as compared to choosing the appropriate guess for the second branch and therefore, the guess would be changing continuously for the second branch until the far-field boundary conditions are not satisfied. According to Weidman [38] and Khan et al. [39], the upper outcome was significantly stable and generally realistic while the lower outcome was not realistic and unstable because we can find here the outcomes for some specific portion of the stretching/stretching parameter. Meanwhile, these results may have no physical significance; they are intriguing from the standpoint of differential equations. When the connected results have further practical value, similar outcomes may occur in other contexts (See Ridha [40]).

### Verification of the Scheme

To show the validation, accuracy, and precision of the scheme, we have prepared the comparison table for the skin friction along the radial direction for some limiting cases. In this comparison, the outcomes of the friction factor along the radial path direction with the various values of α*_A_* (where not including the influence of hybrid nanomaterials, stretching/shrinking parameter, and mass flux parameter) are matched with the available work of Lok et al. [34] (see Table 2). The outcomes of both existing and published are completely matched and look similar up to a five decimal value which shows an excellent sound and harmony. Moreover, this exceptional matching between the two outcomes (present and published) can improve our confidence that our computational unavailable outcomes are accurate.

## 4. Analysis of Results and Discussions

**Figure 2:**
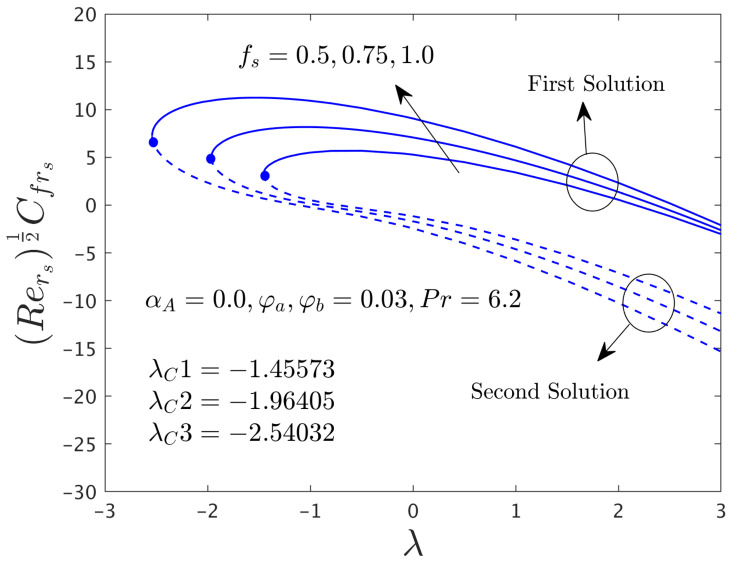
Deviations of ${Re}_{r_{s}}^{0.5}C_{fr_{s}}$ against λ for numerous choices of *f_s_*.

**Figure 3:**
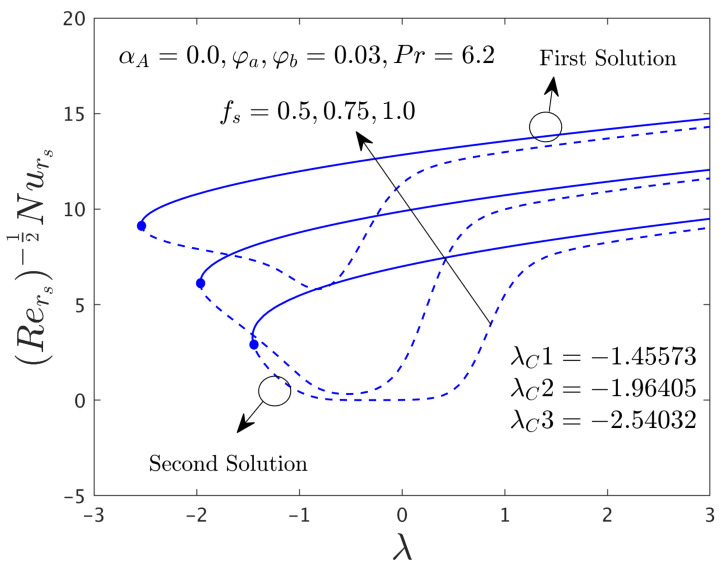
Deviations of ${Re}_{r_{s}}^{-0.5}Nu_{r_{s}}$ against λ for numerous choices of *f_s_*.

**Figure 4:**
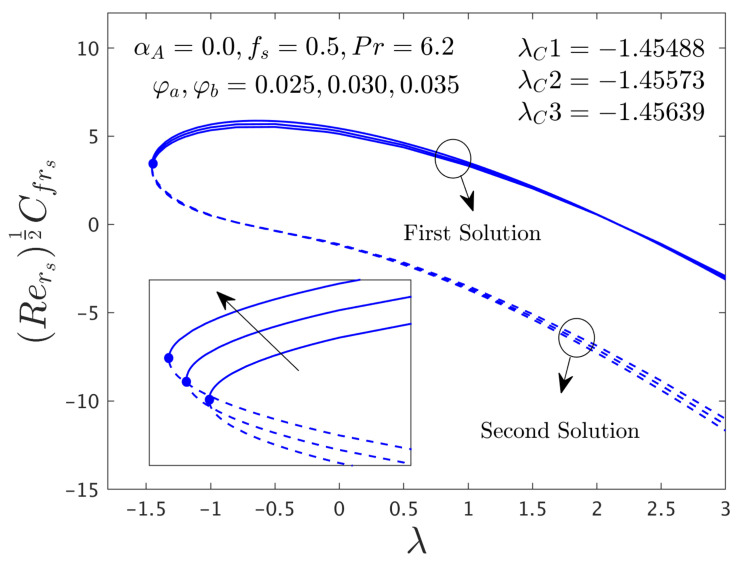
Deviations of ${Re}_{r_{s}}^{0.5}C_{fr_{s}}$ against λ for numerous choices of hybrid nanoparticles.

**Figure 5:**
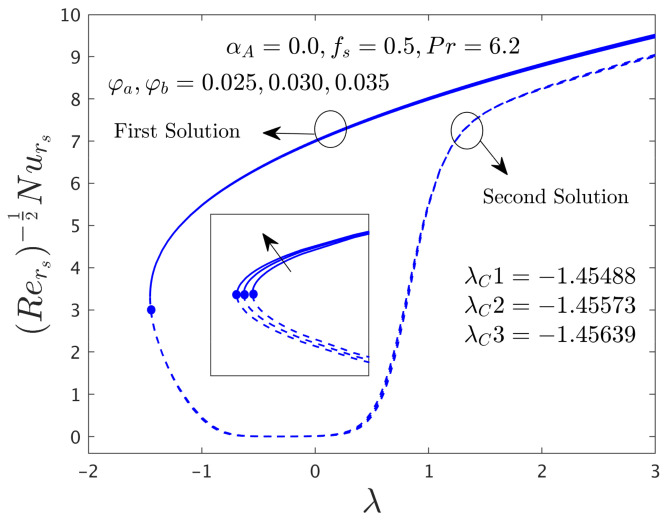
Deviations of ${Re}_{r_{s}}^{-0.5}Nu_{r_{s}}$ against λ for numerous choices of hybrid nanoparticles.

**Figure 6:**
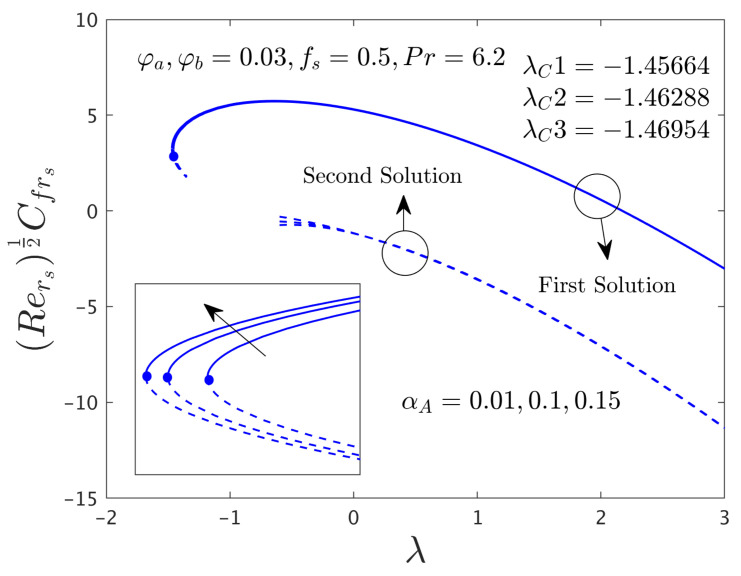
Deviations of ${Re}_{r_{s}}^{0.5}C_{fr_{s}}$ against λ for numerous choices of α*_A_*.

**Figure 7:**
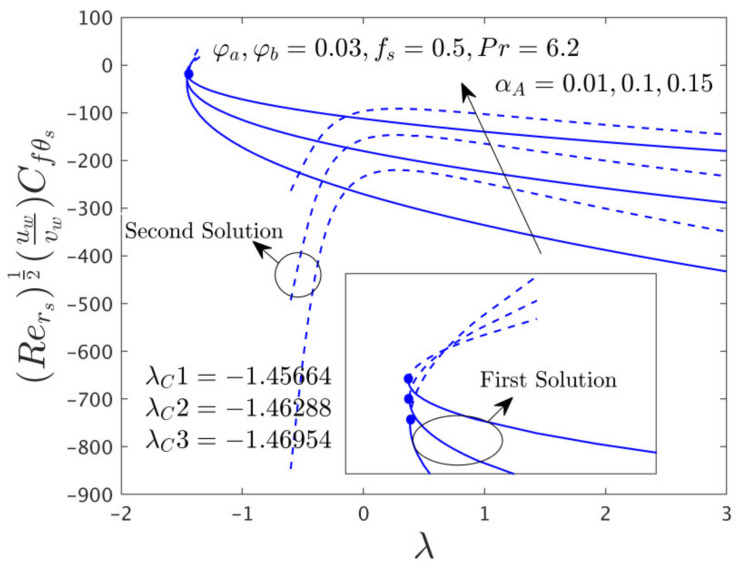
Deviations of ${Re}_{r_{s}}^{0.5}\left(\frac{u_{w}}{v_{w}}\right)C_{f\theta_{s}}$ versus λ for numerous choices of α*_A_*.

**Figure 8:**
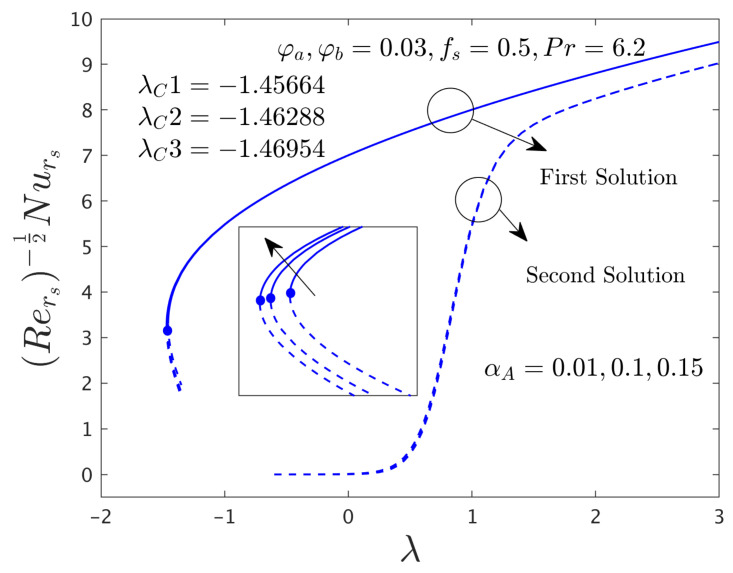
Deviations of ${Re}_{r_{s}}^{-0.5}Nu_{r_{s}}$ against λ for numerous choices of α*_A_*.

**Figure 9:**
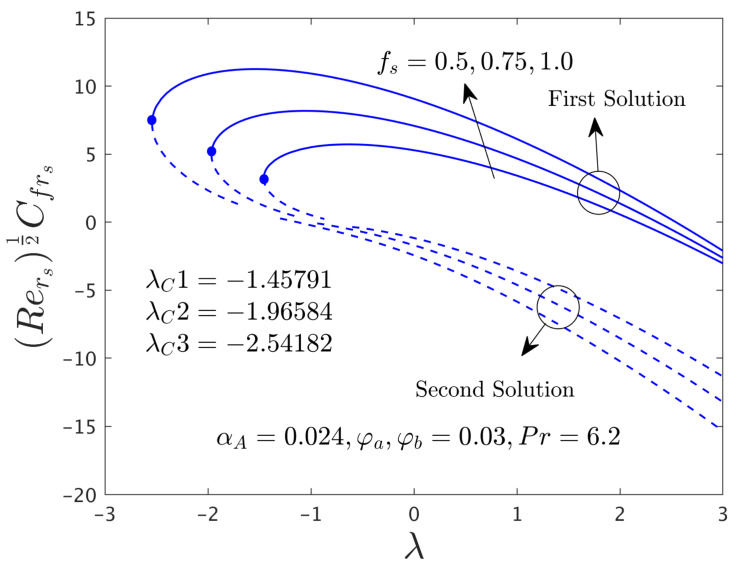
Deviations of ${Re}_{r_{s}}^{0.5}C_{fr_{s}}$ against λ for numerous choices of *f_s_*.

**Figure 10:**
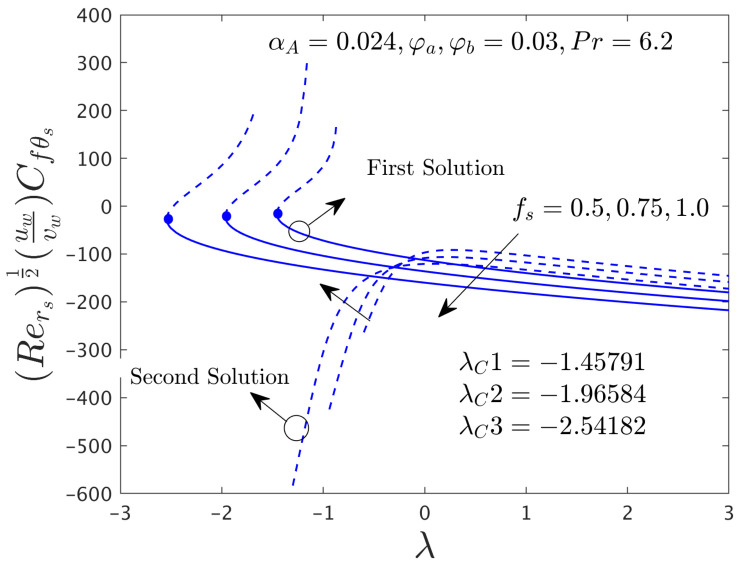
Deviations of ${Re}_{r_{s}}^{0.5}\left(\frac{u_{w}}{v_{w}}\right)C_{f\theta_{s}}$ versus λ for numerous choices of *f_s_*.

**Figure 11:**
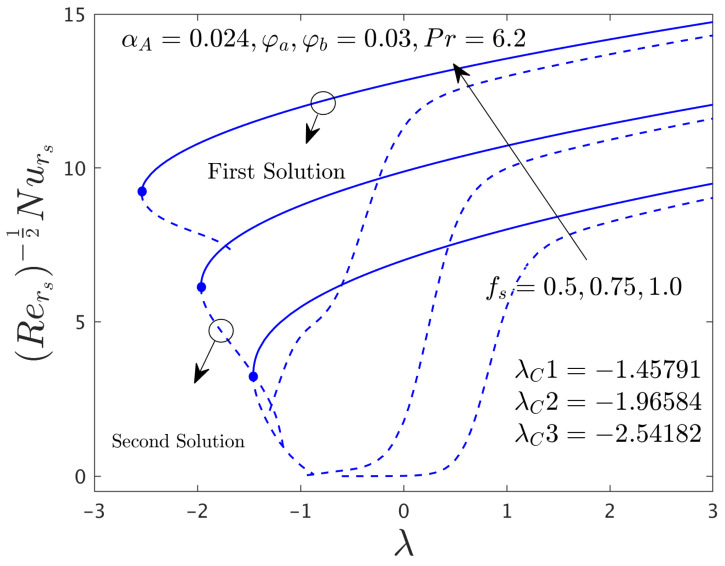
Deviations of ${Re}_{r_{s}}^{-0.5}Nu_{r_{s}}$ against λ for numerous choices of *f_s_*.

**Figure 12:**
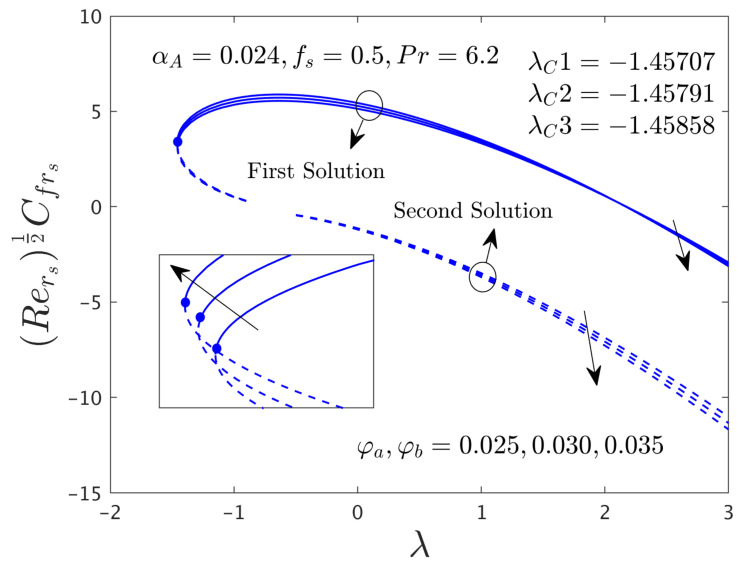
Variations of ${Re}_{r_{s}}^{0.5}C_{fr_{s}}$ against λ for several values of the hybrid nanoparticles.

**Figure 13:**
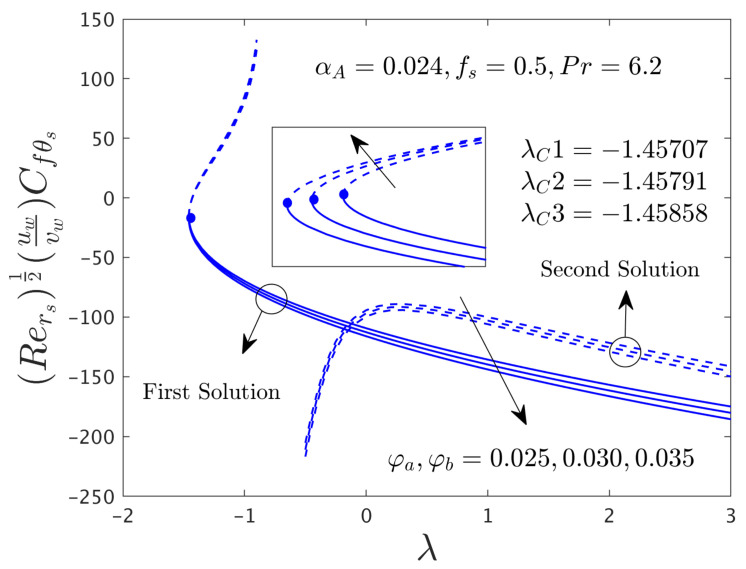
Variations of ${Re}_{r_{s}}^{0.5}\left(\frac{u_{w}}{v_{w}}\right)C_{f\theta_{s}}$ versus λ for numerous choices of the hybrid nanoparticles.

**Figure 14:**
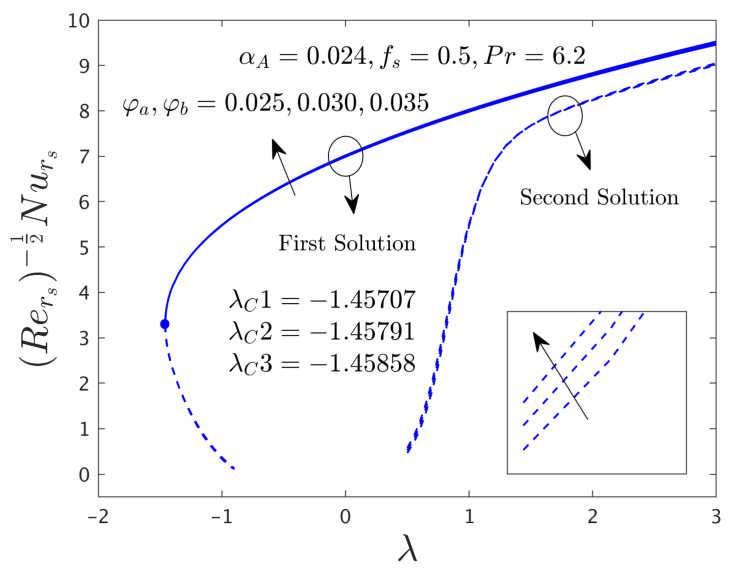
Deviations of ${Re}_{r_{s}}^{-0.5}Nu_{r_{s}}$ against λ for several values of the hybrid nanoparticles.

In this segment of the work, we have highlighted the detailed discussions of the first and second branch outcomes by numerical tables and as well as numerous graphical pictures. The problem comprised the following dimensionless parameters such as the mass flux parameter *f_s_*, the rotation disk parameter α*_A_*, the stretching/shrinking parameter λ, and the hybrid nanoparticles ϕ*_a_* and ϕ*_b_*. The impact of these sundry distinguished parameters on the skin friction coefficients (SFCs) along with the radial and azimuthal directions and local rate of heat transfer of the water-based graphene oxide-molybdenum disulfide hybrid nanomaterials for both dissimilar branches of outcomes are presented in Figure 2, Figure 3, Figure 4, Figure 5, Figure 6, Figure 7, Figure 8, Figure 9, Figure 10, Figure 11, Figure 12, Figure 13 and Figure 14. Moreover, the choices of the SFC in both directions (radial and azimuthal) along with local heat transfer rate are also captured in the tabular form (see Table 3 and Table 4) with the influence of the selected parameters. For simulations, we have fixed the choice of the number for the selected comprised parameters throughout the paper are the following as λ = –1.1, *f_s_* = 0.5, ϕ*_a_* = 0.03 and ϕ*_b_* = 0.03, while it is also written in each window of the graphs. Meanwhile, the paper graphs were drawn for the case of rotating disk parameter α*_A_* = 0.024 and non-rotating disk parameter α*_A_* = 0.0. Note that branches of first and second solution finding for the case of the rotating parameter was quite complex as compared to the case of non-rotating disk parameter, therefore, we have generated here for the first time both branches solution and it was looking too complex from the structure of the picture as well, especially, see the skin friction along with the azimuthal directions. Additionally, the blue solid lines denote the first branch solution and the dash blue lines demonstrate the second branch solution. Also, the point where both solutions are merged and this point is called a bifurcation point.

### 4.1. Analysis of the Constructed Tables

Table 3 and Table 4 display the numerical outputs of the SFCs in the directions of radial and azimuthal and as well as the heat transfer rate of the hybrid (GO-MoS_2_/water) nanoparticles for the consequence of the enormous distinct selected values of the influential parameters when λ = –0.75 and *Pr* = 6.2. Here, the outcomes were spontaneously produced for both solution branches (first solution (FS) and second solution (SS)). The absolute values of the SFCs in both directions and $-\left(k_{hnf}/k_{f}\right)S^{\prime }\left(0\right)$ for first solution branch upsurges with higher values of the volume fraction of hybrid (GO-MoS_2_/water) nanoparticles, ϕ*_a_* and ϕ*_b_* while the drag coefficients for the second solution are similar to the first solution and inverted significantly the behavior of the $-\left(k_{hnf}/k_{f}\right)S^{\prime }\left(0\right)$ for the second solution. The highest consequence values of *f_s_* enriches the heat transfer rate and the absolute values of the SFCs in both directions for the FS while the $\left(\alpha_{A}{}^{-1}\mu_{hnf}/\mu_{f}\right)G^{\prime }\left(0\right)$ and $-\left(k_{hnf}/k_{f}\right)S^{\prime }\left(0\right)$ behavior of the outcome for the second branch are similar as seen in the first solution and reversed the pattern of outcomes for $\left(\mu_{hnf}/\mu_{f}\right)F^{\prime \prime }\left(0\right)$. Moreover, the physical quantities like $\left(\mu_{hnf}/\mu_{f}\right)F^{\prime \prime }\left(0\right)$ and $-\left(k_{hnf}/k_{f}\right)S^{\prime }\left(0\right)$ augmented but reduce the $\left(\alpha_{A}{}^{-1}\mu_{hnf}/\mu_{f}\right)G^{\prime }\left(0\right)$ for the stable outcome with larger values of α*_A_*. Meanwhile, both the SFCs and $-\left(k_{hnf}/k_{f}\right)S^{\prime }\left(0\right)$ behavior of the outcomes are significantly decreased for the SS owing to the amplification in the values of α*_A_*.

### 4.2. For the Example of Non-Rotating Disk Parameter α_A_ = 0.0

Here, in this subsection of the result and discussions, we have to observe the impact of the mass suction parameter *f_s_* and hybrid nanoparticles ϕ*_a_* and ϕ*_b_* on the SFCs both directions and transfer rate of heat of the hybrid (GO-MoS_2_/water) nanoparticles for the first and second solution when the rotating disk parameter is taken to be zero. Note that the drag coefficient in the azimuthal direction $\left(\alpha_{A}{}^{-1}\mu_{hnf}/\mu_{f}\right)G^{\prime }\left(0\right)$ tends to infinity when α*_A_* = 0.0. Therefore, the behavior of the fluid flow in the radial direction and heat transfer characteristics only is captured graphically for the case of a non-rotating parameter.

#### 4.2.1. Consequence of the Mass Flux Parameter (*f_s_*)

Figure 2 and Figure 3 illustrate the consequence of *f_s_* on the $\left(\mu_{hnf}/\mu_{f}\right)F^{\prime \prime }\left(0\right)$ and $-\left(k_{hnf}/k_{f}\right)S^{\prime }\left(0\right)$ of the hybrid (GO-MoS_2_/water) nanoparticles for the stable and unstable solutions against λ, respectively. An augmentation of *f_s_* escalates the SFCs in both directions for FBS and decelerates for the SBS while the rate of heat transfer significantly boosts up in both solution branches. Physically, an improvement in *f_s_* transports the flow of (GO-MoS_2_/water) hybrid nanofluid closer to the surface of the rotating disk which can slow down the profiles of velocity, as a consequence, the SFCs upsurges. In addition, the outcome does not exist for λ < λ*_C_*, non-uniqueness of the outcome is originating for the positive and negative values of λ > λ*_C_*, but the upshot is single for λ = λ*_C_*. Therefore, the outcomes are divided into forks for the case of the shrinking region. For each value of *f_s_* (0.5, 0.75, and 1.0), we have found the following bifurcation values such as −1.45573, −1.96405, and −2.54032, respectively. These critical values are also highlighted in both respective graphs. The magnitude or absolute of the bifurcation value |λ*_C_*| escalates for higher values of *f_s_*, which shows a deceleration in the separation of the boundary layer.

#### 4.2.2. Consequence of Hybrid (GO-MoS_2_/Water) Nanoparticle (ϕ)

The influence of the hybrid nanoparticles (ϕ) on $\left(\mu_{hnf}/\mu_{f}\right)F^{\prime \prime }\left(0\right)$ and $-\left(k_{hnf}/k_{f}\right)S^{\prime }\left(0\right)$ versus λ for branches of FS and SS are exemplified in Figure 4 and Figure 5, respectively. The outcomes of Figure 4 display that the $\left(\mu_{hnf}/\mu_{f}\right)F^{\prime \prime }\left(0\right)$ rises for both dissimilar branch answers due to escalations of ϕ*_a_*, ϕ*_b_* and then it is slightly decreased for the specific portion against λ in the stable branch solution. The existence of the solution sets in the current figures is the same as mentioned in Figure 2 and Figure 3. On the other hand, the heat transfer rate (see Figure 5) for the stable outcome improvements and moderates for SBS in response to the larger values of the hybrid nanoparticles. In a general situation, the hybrid nanoparticles have a direct relation with the heat transfer rate. Therefore, the increasing values of hybrid nanoparticles can develop the impact of thermal conductivity and as a response; this higher conductivity can grow up the heat transfer rate. Moreover, the bifurcation number of points like −1.45488, −1.45573, and −1.45639 are calculated in response to the three distinct values of the hybrid nanoparticles (0.025, 0.030, and 0.035), respectively. From these three bifurcation numbers of values, we can see a pattern that the magnitude of the critical number rises with the continuous forward values of the hybrid nanoparticles. In regards to this behavior, the separation of the boundary layers brings down with improving values of ϕ*_a_* and ϕ*_b_*.

### 4.3. For the Example of Rotating Disk Parameter, α_A_ ≠ 0.0

This research investigates the influence of the comprised selected influential parameters on gradients of the SFCs and as well as the $-\left(k_{hnf}/k_{f}\right)S^{\prime }\left(0\right)$ of the hybrid (GO-MoS_2_/water) nanoparticles against λ for the distinct branch solutions when α*_A_* ≠ 0.0. For the case of the non-zero rotating disk parameter, the behavior of the fluid flow in the radial and azimuthal directions along with heat transfer characteristics are captured in Figure 6, Figure 7, Figure 8, Figure 9, Figure 10, Figure 11, Figure 12, Figure 13 and Figure 14. In addition, the non-uniqueness of the outcome is found for the case of (λ > λ*_C_*), and no outcome is seen for the case of (λ < λ*_C_*), whereas, the outcome is unique or individual for the case of λ = λ*_C_*.

#### 4.3.1. Consequence of the Rotating Disk Parameter (α*_A_*)

Figure 6, Figure 7 and Figure 8 display the variation of α*_A_* on both direction of the SFCs and $-\left(k_{hnf}/k_{f}\right)S^{\prime }\left(0\right)$ of the hybrid (GO-MoS_2_/water) nanomaterials against λ for the branch of FS and as well as SS, respectively. From these pictures, it is observed that $\left(\mu_{hnf}/\mu_{f}\right)F^{\prime \prime }\left(0\right)$ and $-\left(k_{hnf}/k_{f}\right)S^{\prime }\left(0\right)$ upsurges for the FBS and reduces for the SBS due to the larger values of α*_A_*, while the $\left(\alpha_{A}{}^{-1}\mu_{hnf}/\mu_{f}\right)G^{\prime }\left(0\right)$ enriches for both branches. Physically, the azimuthal velocity deceleration is caused by the superior impacts of α*_A_*, which is reasonably transferred to the next adjacent hybrid nanofluid layers. Consequently, the SFC increases in the corresponding azimuthal direction. Moreover, the first solution is smoothly drawn against λ for the varying selected parameter while the breakdown is occurred at the second branch owing to the non-zero α*_A_*. In these graphs, the second branch solution is terminated or broken at some finite value λ_0_ of λ. In other words, the graph for the second solution is broken in the range of –1.35 < λ < –0.6 for the varying choices of α*_A_*. Besides, the larger values of α*_A_* signify that the magnitude of the bifurcation values increases (see the window of Figure 6, Figure 7 and Figure 8) which demonstrates to slow down the boundary layer separations.

#### 4.3.2. Consequence of the Mass Flux Parameter (*f_s_*)

The deviations of the SFCs in both distinct directions and $-\left(k_{hnf}/k_{f}\right)S^{\prime }\left(0\right)$ of the hybrid (GO-MoS_2_/water) nanoparticles for two different solutions with *f_s_* are highlighted in Figure 9, Figure 10 and Figure 11, respectively. In radial path the SFC upsurges for the FS and reduces for the SS due to higher influences of mass suction parameter *f_s_*, while the SFC in the azimuthal path shrinkages. In fact, the behavior of the outcomes for SFC in the radial path shown in Figure 9 looks similar to Figure 2 but here only a breakdown is appeared in the second branch due to the case α*_A_* ≠ 0.0. With this non-zero value of α*_A_*, we have constructed Figure 10 where the behavior of the fluid flow in the azimuthal direction was different as compared to other figures of the physical quantities. On the other hand, the heat transfer rate progresses for two different solutions because of the superior impacts of *f_s_* as shown schematically in Figure 11. In comparison, the magnitude of the bifurcation values of the shear stress in the radial direction and heat transfer rate when α*_A_* = 0.024 is slightly higher than the critical values found in Figure 2 and Figure 3, respectively, for the superior values of *f_s_*. This shows that the separation of the boundary layers was better in the case of the rotating disk parameter as compared to the non-rotating disk parameter.

#### 4.3.3. Consequence of Hybrid (GO-MoS_2_/Water) Nanoparticle (ϕ)

The SFCs in both directions and $-\left(k_{hnf}/k_{f}\right)S^{\prime }\left(0\right)$ of the hybrid (GO-MoS_2_/water) nanoparticles are exemplified in Figure 12, Figure 13 and Figure 14, respectively, for various distinct values of ϕ*_a_* and ϕ*_b_*. It can be remarked from these figures that the multiple (FB and SB) solutions exist for the cases of shrinking and stretching parameter. Outcomes divulge that in the radial path the SFC and $-\left(k_{hnf}/k_{f}\right)S^{\prime }\left(0\right)$ for both dissimilar branches of outcome upsurges with ϕ*_a_* and ϕ*_b_*, while the in the azimuthal path the SFC $\left(\alpha_{A}{}^{-1}\mu_{hnf}/\mu_{f}\right)G^{\prime }\left(0\right)$ declines for both branches. Meanwhile, the $\left(\alpha_{A}{}^{-1}\mu_{hnf}/\mu_{f}\right)G^{\prime }\left(0\right)$ near the bifurcation values upsurges with higher hybrid nanoparticles. In general explanations, the hybrid nanoparticles can bring the fluid particles near the surface of the disk due to the presence of suction and rotating parameters, therefore, the motion of the fluid was stopped or very negligible. As a consequence, the friction factor boosted up with the influence of hybrid nanoparticles. Moreover, in these figures, the magnitude of the critical values is little advance for the dissimilar values of hybrid nanoparticles as compared to the graphs drawn (see Figure 4 and Figure 5) for the case of α*_A_* = 0.0.

## 5. Conclusions

We have investigated the axisymmetric rotational stagnation-point Agrawal flow of a water-based molybdenum disulfide-graphene oxide hybrid nanoparticles and heat transfer characteristics on a radially permeable shrinking or stretching rotating disk. By executing the pertinent self-similarity transformation, the given problem reduced to a set of similarity Equations (10)–(13), identifying the four dissimilar influential parameters i.e., *f_s_* the mass flux velocity, α*_A_* the dimensionless rotating disk parameter, λ the stretching/shrinking parameter, and ϕ called the volume fraction of nanoparticles. Outcomes divulged that multiple branch solutions appeared for both cases of the stretching/shrinking parameter, whereas, we have supplementary assumed to start our numerical computations for the two distinct cases α*_A_* = 0 and α*_A_* = 0.024. Therefore, the consequences of the comprised different dimensionless distinguished parameters for the cases of α*_A_* = 0 and α*_A_* = 0.024 on the physical quantities were premeditated by numerous graphs and tables. The main findings of the considered simulations are summarized as follows:

- The shear stress in the radial direction $\left(\mu_{hnf}/\mu_{f}\right)F^{\prime \prime }\left(0\right)$ upsurges for the first solution but reduces for the second solution with higher values of *S* when α*_A_* = 0, *Pr* = 6.2, ϕ*_a_* = 0.03 and ϕ*_b_* = 0.03 while the $-\left(k_{hnf}/k_{f}\right)S^{\prime }\left(0\right)$ significantly rises.
- The influence of hybrid nanoparticles on heat transfer rate $-\left(k_{hnf}/k_{f}\right)S^{\prime }\left(0\right)$ suggests an improvement for FB outcome but decays for the SB outcome when α*_A_* = 0, *Pr* = 6.2 and *f_s_* = 0.5 while the SFC in the direction of radial enriches for two solution branches.
- The rate of heat transfer $-\left(k_{hnf}/k_{f}\right)S^{\prime }\left(0\right)$ and the $\left(\mu_{hnf}/\mu_{f}\right)F^{\prime \prime }\left(0\right)$ augments for the first solution and declines for the second solution with higher values of α*_A_* while the shear stress in the azimuthal direction $\left(\alpha_{A}{}^{-1}\mu_{hnf}/\mu_{f}\right)G^{\prime }\left(0\right)$ improves for two dissimilar branch outcomes.
- The multiple branch outcomes exist for both cases of stretching (λ > 0) and shrinking (λ < 0), whereas, a solution is unique for (λ = λ*_C_*) and no solution found for λ < λ*_C_*. Also, the full line and broken line are generated for the SBS when α*_A_* = 0 and α*_A_* = 0.024, respectively, versus the stretching/shrinking parameter λ owing to the selected comprised dimensionless parameters.
- For the variation of the selected influential parameters such as mass flux parameter *S* and hybrid nanoparticles ϕ*_a_* and ϕ*_b_*, the magnitude or absolute of the bifurcation values are sophisticated for the case of α*_A_* = 0.024 as compared to the case of α*_A_* = 0.0.

## Figures and Tables

**Table 1:** The thermophysical properties of the (GO + MoS_2_)-H_2_O hybrid nanoparticles.

Properties	$\rho \left(kg/m^{3}\right)$	$c_{p}\left(J/\left(kg.K\right)\right)$	$k\left(W/\left(m.K\right)\right)$	*Pr*
water	997.1	4179	0.613	6.2
GO	1800	717	5000	-
MoS_2_	5060	397.21	904.4	-

**Table 2:** A judgment of $F^{\prime \prime }\left(0\right)$ with Lok et al. [34] for dissimilar selected values of α*_A_* when ϕ*_a_* = ϕ*_b_* = f*_s_* = 0 and λ = 0.

α*_A_*	Present Results
0	2.0000000
25	6.8191539
100	17.055614
225	30.488228
400	46.443400
625	64.560920
900	84.604186

**Table 3:** Computational data of the SFCs along the radial and azimuthal directions for the several values of the selected parameters when λ = –0.75 and *Pr* = 6.2.

ϕ*_a_*, ϕ*_b_*	*f_s_*	α*_A_*	$\left(\mu_{hnf}/\mu_{f}\right)F^{\prime \prime }\left(0\right)$		$\left(\alpha_{A}{}^{-1}\mu_{hnf}/\mu_{f}\right)G^{\prime }\left(0\right)$	
			First Solution	Second Solution	First Solution	Second Solution
0.030	0.5	0.025	5.48360000	−0.30821143	−77.457484	−321.05271
0.034	-	-	5.58925670	−0.31700310	−78.899781	−327.49352
0.038	-	-	5.69667990	−0.32631734	−80.359778	−334.07201
0.030	0.5	-	5.48360000	−0.30821143	−77.457484	−321.05271
-	0.6	-	6.35703390	−0.28259111	−87.837482	−313.34468
-	0.7	-	7.24986100	−0.31207114	−97.893453	−289.31890
0.030	0.5	0.05	5.49047030	−0.40387954	−38.740770	−122.06876
-	-	0.10	5.50419650	−0.56038023	−19.382388	−47.018143
-	-	0.15	5.51790350	−0.70282081	−12.929571	−27.155183

**Table 4:** Computational data of the Nusselt number for the numerous choice of the dissimilar comprised parameters when λ = –0.75 and *Pr* = 6.2.

ϕ*_a_*, ϕ*_b_*	*f_s_*	α*_A_*	$\left(\mu_{hnf}/\mu_{f}\right)F^{\prime \prime }\left(0\right)$	
			First Solution	Second Solution
0.030	0.5	0.025	5.81200290	0.00001712
0.034	-	-	5.81058090	0.00002175
0.038	-	-	5.80924700	0.00002739
0.030	0.5	-	5.81200290	0.00001712
-	0.6	-	7.02627280	0.00079153
-	0.7	-	8.23881650	0.01663883
0.030	0.5	0.05	5.81281430	0.00000319
-	-	0.10	5.81443370	0.00000021
-	-	0.15	5.81604840	0.00000002

## Data Availability

Not applicable.

## Nomenclature

*F*(*ξ*), *G*(*ξ*)	Dimensionless velocity stream function
*c_p_*	Specific heat at constant pressure (J/kg K)
${Re}_{r_{s}}$	Local Reynolds number
$Nu_{r_{s}}$	Local Nusselt number
*P_s_*	Pressure (kg/m s^2^)
$C_{f\theta_{s}}$	Skin friction coefficient along the azimuthal direction
(*u_s_*, *v_s_*, *w_s_*)	Velocity components (m/s)
(*u_E_*, *v_E_*, *w_E_*)	Free-stream velocities (m/s)
*w_a_*	Constant mass flux velocity (m/s)
*Pr*	Prandtl number
*T_s_*	Temperature of the fluid (K)
*f_s_*	Mass suction parameter
*u_w_*	Surface velocity of the disk (m/s)
$C_{fr_{s}}$	Skin friction coefficient along the radial direction
*T_w_*	Constant surface temperature (K)
(*r_s_*, *θ_s_*, *z_s_*)	Cylindrical coordinate system (m)
*a*	Constant parameter having units (m s)^−1^
*T*_∞_	Constant ambient temperature (K)
*v_w_*	Rotating velocity of the disk (m/s)
*k*	Thermal conductivity (W/(m K))
*S*(*ξ*)	Dimensionless tempetature
***Greek symbols***	
α	Thermal diffusivity (m^2^/s)
α	*_A_*Rotating disk parameter
λ	Stretching/Shrinking parameter
μ	Absolute viscosity (N s/m^2^)
ν*_f_*	Kinematic viscosity (m^2^/s)
*ξ*	Pseudo-similarity variable
ρ	Density (kg/m^3^)
ϕ	Solid volume fraction of nanoparticles
ψ	Stream function
ω	*_s_*Constant angular velocity (s^−1^)
***Acronyms***	
GO	Graphene oxide
PDEs	Partial differential equations
SFCs	Skin friction coefficients
FS	First solution
H_2_O	Water
	bvp4cBoundary value problem of the fourth-order
SS	Second solution
MoS_2_	Molybdenum disulfide
FBS	First branch solution
BCs	Boundary conditions
SBS	Second branch solution
ICs	Initial conditions
***Subscripts***	
*w*	Wall boundary condition
*hnf*	Hybrid nanofluid
*f*	Working base fluid
a, b	Hybrid nanoparticles (GO and MoS_2_)
∞	Far-field condition
***Superscript***	
′	Differentiation with respect to *ξ*
